# EYA2 promotes lung cancer cell proliferation by downregulating the expression of PTEN

**DOI:** 10.18632/oncotarget.22860

**Published:** 2017-12-02

**Authors:** Zhaoming Li, Ran Qiu, Xia Qiu, Tian Tian

**Affiliations:** ^1^ Department of Oncology, The First Affiliated Hospital of Zhengzhou University, Zhengzhou, China; ^2^ Wuhan Institute of Bioengineering, Wuhan, China; ^3^ Department of Medicine, Shangqiu Medical School, Shangqiu, China; ^4^ Department of Neurology, The First Affiliated Hospital of Zhengzhou University, Zhengzhou, China

**Keywords:** EYA2, PTEN, lung cancer, cell proliferation, prognosis

## Abstract

Lung cancer is the leading cause of cancer-related death worldwide. Despite advances have been made in diagnosis and therapeutic strategies, the prognosis of lung cancer is still very poor. Eyes absent transcriptional cofactor EYA2 has been shown to promote lung cancer cell growth, however, the underlying molecular mechanism is still not fully understood. In the present study, we found that EYA2 was up-regulated in lung cancer, and EYA2 led to increased cell proliferation by inhibiting Phosphatase and tensin homologue (PTEN) expression via modulation of miR-93. Additionally, survival analysis showed that lung cancer patients with higher EYA2 expression predicted a worse prognosis. Therefore, these findings demonstrate that EYA2 may play an important role in lung cancer occurrence and progression. Targeting EYA2 may provide a feasible approach in developing novel anticancer therapeutics.

## INTRODUCTION

Lung cancer is the deadliest malignancy accounting for nearly 1 of every 4 cancer-related deaths worldwide [1–4]. It is typically divided into small-cell lung carcinoma or non-small cell lung carcinoma (NSCLC), which account for ∼20% and ∼80% of all lung carcinomas, respectively [5, 6]. Despite significant progress has been made in diagnosis and therapeutic strategies, the five-year overall survival rate of lung cancer patients is still dismal for all stages. This is basically due to the lack of early diagnostic tools of lung cancer and its underlying pathogenesis mechanisms is still not fully understood until now [7–9]. Therefore, understanding molecular changes involved in the development of lung tumor is essential for the identification of novel anticancer therapeutic targets.

The eyes absent (EYA) family proteins (EYA1-4) are highly conserved transcriptional cofactors and important components of Retinal Determination Gene Network (RDGN) signaling [10–13]. Recent studies indicate that EYA family proteins contribute to tumor initiation and progression in various cancers. EYA2 was reported to be up-regulated in epithelial ovarian cancer and promotes tumor growth [14]. EYA2 was required to mediate the pro-metastatic functions of Six1 in breast cancer [15]. The EYA2 is critical for PLZF-RARA-induced leukemogenesis [16]. Moreover, EYA2 has been shown to promote cell growth in lung cancer [17]. EGFR/miR-338-3p/EYA2 axis controls breast tumor growth and lung metastasis [18]. Overexpression of miR-30a inhibits migration and invasion via targeting EYA2 in lung cancer [19]. However, the underlying regulatory mechanisms by which this occurs have not been fully elucidated.

Phosphatase and tensin homologue (PTEN) was reported to act as a tumor suppressor by inhibiting PI3K pathway activation in various cancers [20]. PTEN protein expression is downregulated in well to moderately differentiated NSCLC tumors [21, 22]. Previous studies suggested that dysregulation of PTEN may cause tyrosine kinase inhibitors resistance in NSCLC patients [23, 24]. Moreover, NSCLC patients with low PTEN expression had worse prognosis than those patients with higher PTEN expression [25].

In this study, the increased expression of EYA2 was detected in lung cancer. Forced expression of EYA2 could enhance lung cancer cell growth both *in vivo* and *in vitro*. Furthermore, EYA2 could inhibit the expression of PTEN via modulation of miR-93 in lung cancer cells. Additionally, survival analysis showed that lung cancer patients with higher expression of EYA2 had a worse overall survival.

## RESULTS

### EYA2 is up-regulated in lung cancer

**Figure 1:**
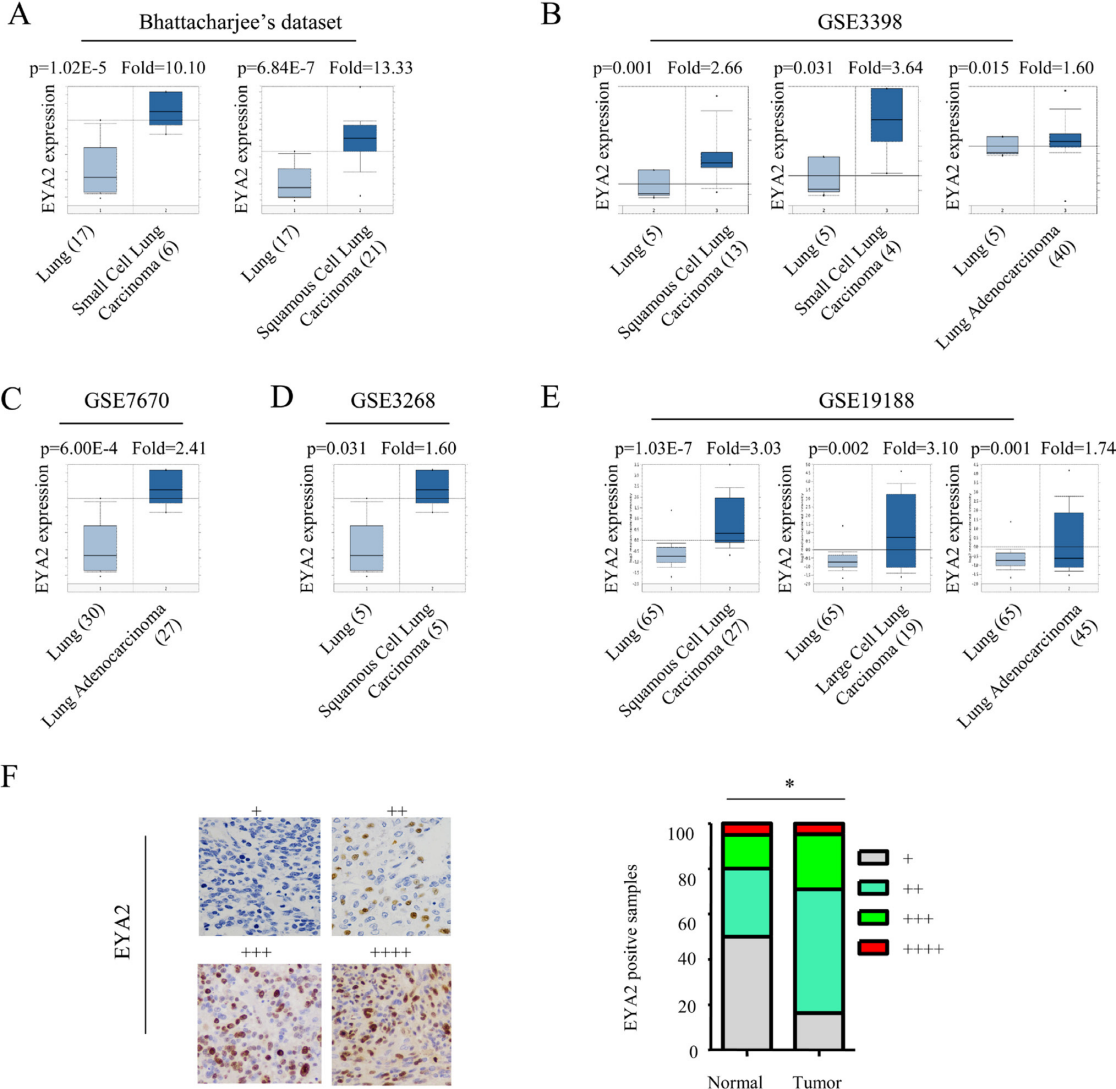
EYA2 is overexpressed in lung cancer (**A**–**E**) Expression levels of EYA2 mRNA in five lung cancer datasets: Bhattacharjee’s dataset (A), GSE3398 (B), GSE7670 (C), GSE3268 (D) and GSE19188 (E). (**F**) EYA2 expression in 86 lung cancer tissues and 20 non-tumor lung tissues examined by immunohistochemisty. Expression levels of EYA2 were scored semi-quantitatively based on the percentage of positive cells according to the following scale: +, <25%; ++, 25–49%; +++, 50–74%; and ++++, 75–100% (G). Scale bars = 50 µm.

To evaluate the role of EYA2 in lung cancer development, we first examined the mRNA expression levels of EYA2 in five lung cancer datasets (Bhattacharjee’s dataset, GSE3398, GSE7670, GSE3268 and GSE19188) using ONCOMINE database [26]. In the microarray gene expression studies, EYA2 mRNA levels in lung tumor tissues were significantly higher than those in the non-tumor lung tissues, the EYA2 mRNA levels ranged from 1.6- to 13.3-fold increase in lung cancer (Figure 1A–1E). In the Bhattacharjee’s dataset, mRNA levels of EYA in small cell lung carcinoma (*n =* 6) and squamous cell lung carcinoma (*n =* 21) were increased respectively by 10.10-fold and 13.33-fold compared with non-tumor lung tissue (*n =* 17). In the GSE3398 dataset, EYA mRNA expression levels in squamous cell lung carcinoma (*n =* 13), small cell lung carcinoma (*n =* 4) and lung adenocarcinoma (*n =* 40) were increased significantly to 2.66-, 3.64- and 1.66-fold of the control, respectively. In the GSE7670 dataset, the mRNA expression level of EYA in lung carcinoma (*n =* 27) was increased by 2.41-fold. In the GSE3268 dataset, EYA mRNA expression level in squamous cell lung carcinoma (*n =* 5) was increased by 1.60-fold. In the GSE19188 dataset, mRNA levels of EYA in squamous cell lung carcinoma (*n =* 27), large cell lung carcinoma (*n =* 19) and lung adenocarcinoma (*n =* 45) were increased to 3.03-, 3.10- and 1.74-fold of the control, respectively. Then, the EYA2 expression in 86 lung cancer tissues and 20 non-tumor lung tissues was examined using immunohistochemisty. The results showed that expression of EYA2 was significantly higher in tumor tissues than in adjacent non-tumor tissues (^*^p < 0.05, Figure 1F). We further analyzed EYA2 expression according to the histological subtype of the lung cancer tissues. As shown in Supplementary Figure 1, the EYA2 expression was upregulated in both lung adenocarcinoma (*p* = 0.012) and lung squamous cell carcinoma (*p* = 0.005), which was also consistent with results from analysis of different lung cancer datasets. Together, these data suggested that EYA2 was up-regulated in lung cancer.

### EYA2 promotes lung cancer cell proliferation *in vitro* and *in vivo*

**Figure 2:**
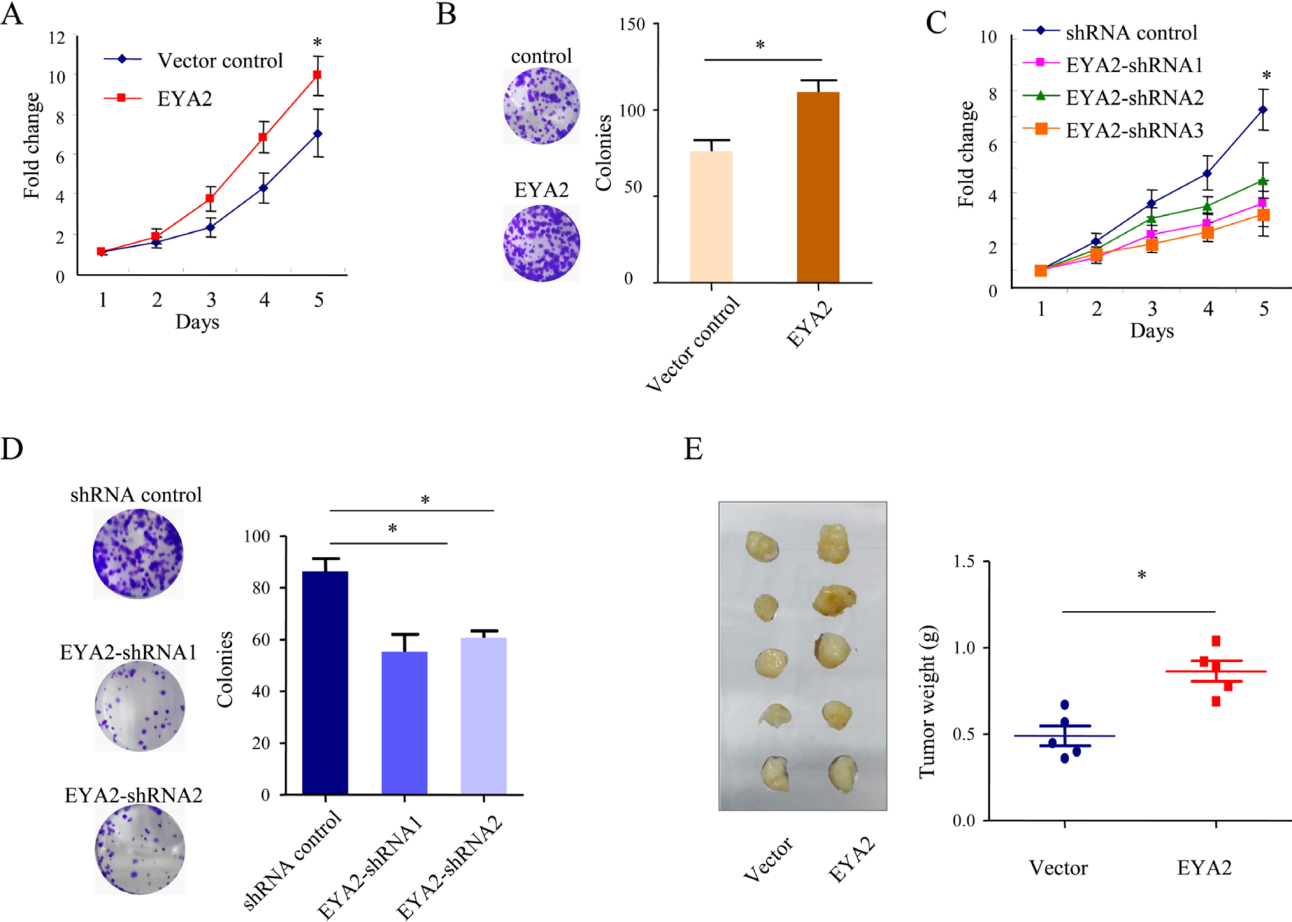
EYA2 promotes lung cancer cell proliferation *in vitro* and *in vivo* (**A**, **B**) cell growth assay (A) and colony formation assay (B) of A549 cells with stable EYA2 expression. (**C**, **D**) cell growth assay (C) and colony formation assay (D) of A549 cells stably transfected with EYA2 shRNAs (EYA2-shRNA1, EYA2-shRNA2 and EYA2-shRNA3). All experiments were performed at least three times; bars, s.e.m.; ^*^p < 0.05. (**E**) *in vivo* xenograft model of lung tumor. A549 cells with stable expression of EYA2 or vector control were subcutaneously injected into two groups of nude mice. Representative images showing ablation of subcutaneous xenograft tumors and the weight of the tumors. Bars, s.e.m.; ^*^p < 0.05.

To address the functional consequence of EYA2 upregulation in lung cancer, we examined the effect of EYA2 on the proliferation of A549 cells. First, we established A549 cells with stable expression of either EYA2 or vector control plasmids. Cell proliferation was then assessed by cell growth assay and colony formation assay. The cell growth assay indicated that the proliferation rate of A549 lung cancer cells overexpressed with EYA2 was significantly higher than that of vector control cells at 5 days after plating (^*^p < 0.05, Figure 2A). The colony formation assay showed that the numbers of colonies of vector control and EYA2 group were 76.0 ± 6.4 and 110.3 ± 6.9, respectively (^*^p = 0.02, Figure 2B). We further tested whether EYA2 was required for the proliferation of A549 cells. The endogenous expression of EYA2 was silenced by lentivirus-mediated shRNA interference. As shown in Figure 2C, the cell growth was obviously suppressed after knockdown of endogeous EYA2 by three different lentivirus-mediated shRNAs in A549 cells. Similarly, the colony number of EYA2-knockdown cells was significantly lower than the number of A549 cells transfected with shRNA control (shRNA control, EYA2-shRNA1 and EYA2-shRNA2, 86.3 ± 4.9, 55.3 ± 6.7 and 60.6 ± 2.6, respectively; ^*^p = 0.005, Figure 2D). We examined the role of EYA2 in another lung cancer cell line H1975. As shown in Supplementary Figure 3, overexpression of EYA2 significantly promotes the cell proliferation of H1975 cells (Supplementary Figure 3A and 3B; ^*^p < 0.05). Furthermore, we evaluated the effects of EYA2 overexpression in a PTEN null cell line H1650. To determine whether EYA2 affect the cell death of lung cancer, we knocked down the endogenous EYA2 in A549 cells and then performed Annexin/PI staining assay. As shown in Supplementary Figure 2, the percentage of apoptotic cells in EYA2 shRNAs groups were significantly increased compared to the scrambled control group (^*^p < 0.05). It indicated that the inhibition of EYA2 promoted the apoptosis of the A549 cells, which was also consistent with previous studies [17]. Taken together, these results indicated that EYA2 promoted lung cancer cell proliferation *in vitro*.

Next, the above findings were further confirmed *in vivo* in xenograft tumor model. A549 cells stably overexpressing EYA2 or vector alone were injected subcutaneously into two groups of nude mice (*n* = 5). Four weeks after injection, the tumors were resected. The mean tumor weight in the group overexpressing EYA2 (0.86 ± 0.06 g) was significantly higher compared to that in the control group (0.49 ± 0.05 g; ^*^p < 0.05, Figure 2E) at the end of the experiment. These results suggested that up-regulated EYA2 promoted the proliferation of lung cancer cells *in vivo*.

### EYA2 represses PTEN expression in lung cancer cells

**Figure 3:**
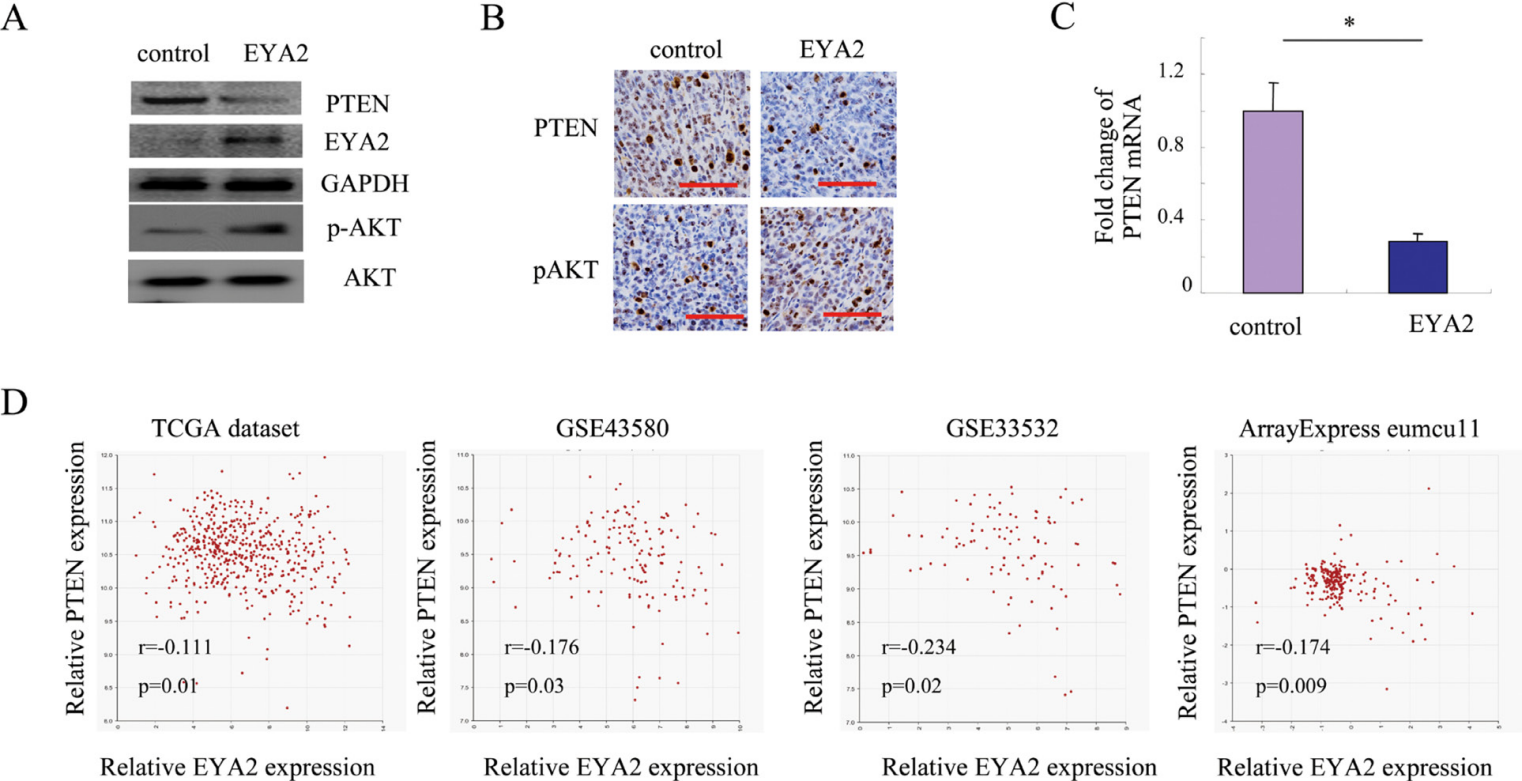
EYA2 represses PTEN expression in lung cancer cells (**A**) The changes of PTEN protein level affected by EYA2 in A549 cells. (**B**) The expression of PTEN and p-AKT in xenografts tumor samples examined by immunochemistry. Scale bars = 50 µm. (**C**) The changes of PTEN mRNA level affected by EYA2 in A549 cells. (**D**) Analysis of the relationship between EYA2 and PTEN using the gene correlation module of the R2 microarray analysis and visualization platform in four independent lung cancer microarray datasets. Bars, s.e.m.; ^*^p < 0.05.

PTEN has been validated as a critical candidate of lung cancer tumor suppressor [27, 28]. In this study, we found that the expression of PTEN could be inhibited by EYA2 in lung cancer cells. Western blot assay showed that PTEN protein levels were dramatically reduced in A549 cells transduced with EYA2 compared with the respective vector control (Figure 3A). Furthermore, we examined PTEN and p-AKT levels in xenograft tumor tissues. As shown in Figure 3B, the level of PTEN in tumor samples with EYA2 overexpression was much lower than that in samples with vector control. Consequently, the level of p-AKT in EYA2 group was much higher compared with the vector control group. To determine whether EYA2 regulates the mRNA expression of PTEN, we analyzed PTEN expression by quantitative PCR analysis. The results showed that the expression of PTEN mRNA in A549 cells transduced with EYA2 decreased to nearly one third of the PTEN mRNA level in the vector control (^*^p < 0.05, Figure 3C).

Next, to confirm these above findings, we performed the co-expression analysis of EYA2 and PTEN in four independent lung cancer microarray datasets. A significantly negative correlation between EYA2 and PTEN was confirmed in multiple datasets (TCGA dataset: *r*: −0.111, *p* = 0.01; GSE43580: *r*: −0.176, *p* = 0.03; GSE33532: *r* = −0.234, *p* = 0.02; and ArrayExpress eumcu11 dataset: *r* = −0.174, *p* = 0.009; Figure 3D). We evaluated PTEN expression by immunohistochemistry and analyzed its correlation with EYA2 expression. As shown in Supplementary Table 1, the expression of PTEN was negative correlated with EYA2 expression in lung cancer (Phi = −0.28, *p* = 0.017). Furthermore, in NSCLC, the expression of PTEN was still negative correlated with EYA2 expression (Phi = −0.32, *p* = 0.012). Collectively, the data demonstrated that there was an inverse correlation between EYA2 and PTEN in lung cancer samples.

### EYA2 promotes cell proliferation via suppression of PTEN in lung cancer

**Figure 4:**
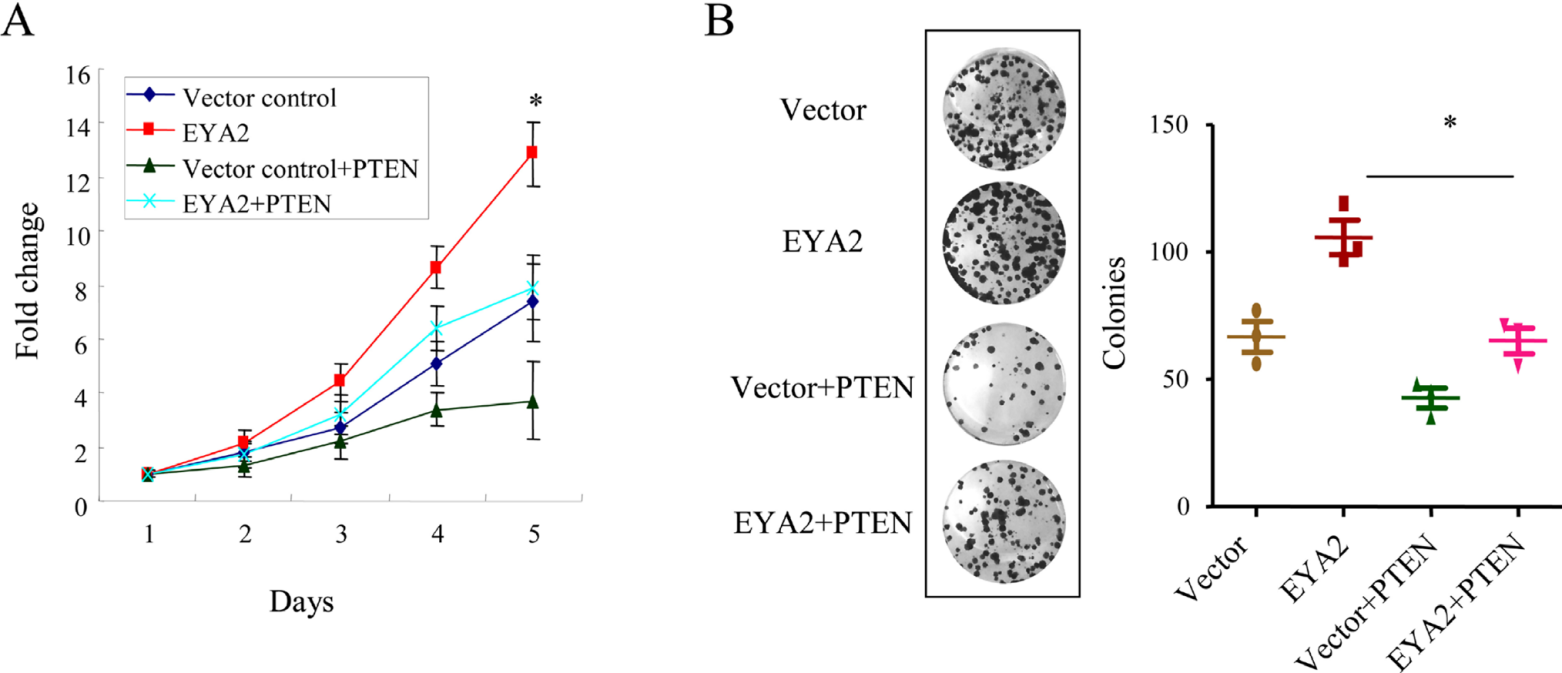
EYA2 enhances cell proliferation through downregulation of PTEN in lung cancer (**A**, **B**) Forced expression of PTEN in A549 cells stably-expressing EYA2, then cell proliferation were examined by cell growth assay (A) and colony formation assay (B). All the experiments were performed in triplicate. Bars, s.e.m.; ^*^p < 0.05.

To address whether EYA2 promotes cell proliferation through suppression of PTEN in lung cancer cells, A549 cells with stable expression of EYA2 were forced to overexpress PTEN. The cell growth assay showed that overexpression of EYA2 enhanced lung cancer cell growth, while re-expression of PTEN significantly compromised the growth advantage conferred by EYA2 in A549 cells (^*^p < 0.05, Figure 4A). Similarly, the colony formation assay also showed that re-expression of PTEN significantly attenuated the growth advantage conferred by EYA2 in A549 cells. The colony numbers of vector control group, EYA2 group and EYA2+PTEN group were (66.6 ± 6.1), (105.7 ± 6.8) and (65.0 ± 5.0), respectively (^*^p < 0.05, Figure 4B). In summary, these results indicated that EYA2 promoted cell proliferation via inhibition of PTEN in lung cancer.

### EYA2 suppresses PTEN expression via modulation of miR-93

**Figure 5:**
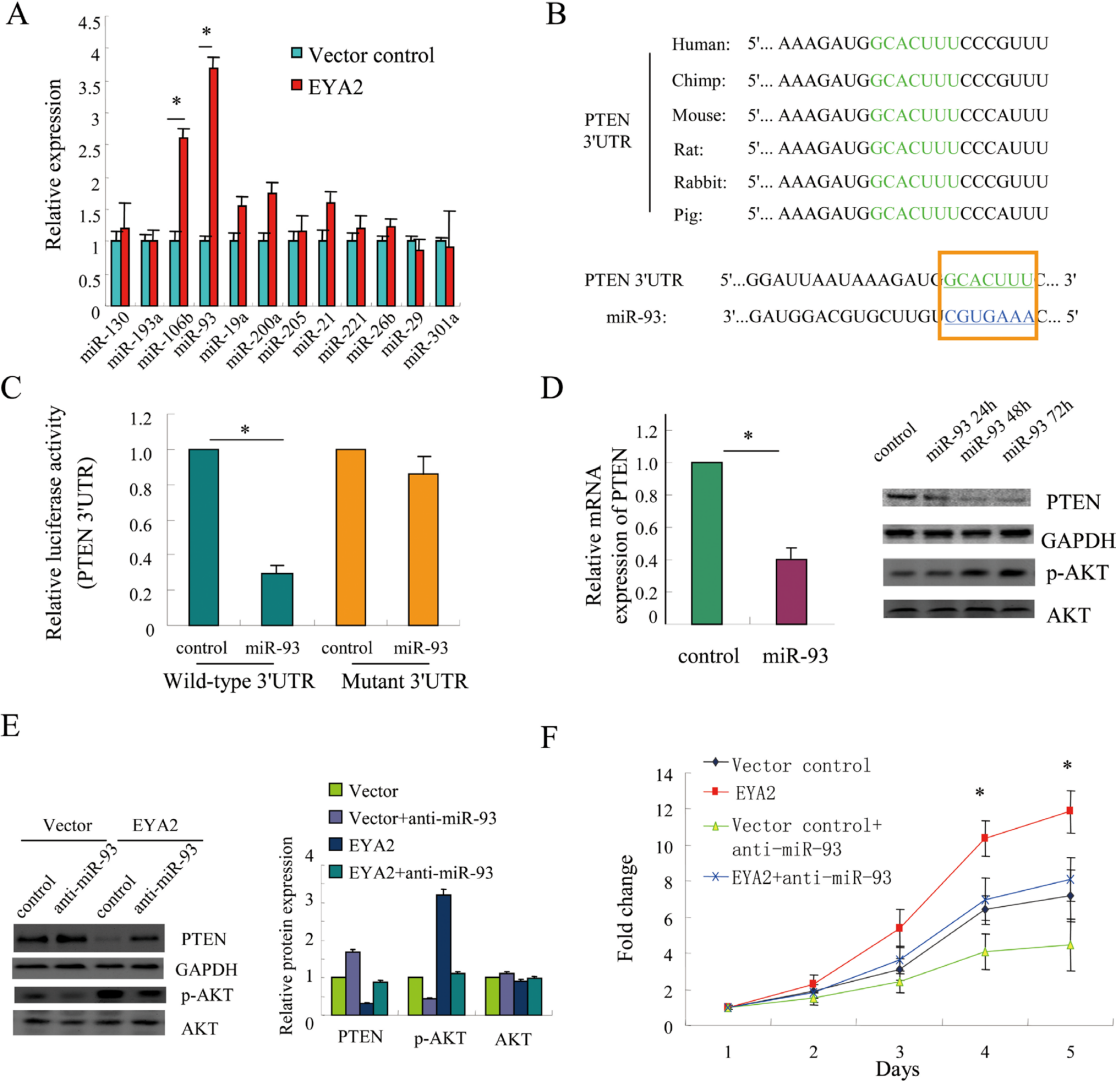
EYA2 suppresses PTEN expression via modulation of miR-93 (**A**) The expression of PTEN-targeting miRNAs affected by EYA2 in A549 cells. (**B**) The predicted miR-93 binding sites in the 3′UTR of PTEN were conserved in different various species. (**C**) A549 cells were transfected with 3′UTR of wild-type and mutated human PTEN promoter reporter (Wild-type 3′UTR or Mutant 3′UTR) in the presence of exogenous miR-93, and luciferase activities were measured using the Dual Luciferase Reporter Assay System (Promega, Madison, WI, USA). (**D**) Forced expression of miR-93 reduced PTEN expression at both the mRNA and protein levels in A549 cells. (**E**) The endogenous miR-93 was suppressed by transfection of A549 cells with anti-miR-93 oligonucleotides, then the suppression effect of EYA2 on PTEN expression was examined by immunoblot assay. The densitometry was quantified with a FluorChem FC2 imaging system (Alpha Innotech, San Leandro, CA, USA). Protein levels were normalized to GAPDH and quantified with respect to vector control group. (**F**) A549 cells with stably-expressing EYA2 were transfected with anti-miR-93 oligonucleotides and cell growth was examined by CCK-8 assay. All the experiments were performed in triplicate. Bars, s.e.m.; ^*^p < 0.05.

It has been estimated that PTEN can be targeted by multiple miRNAs, such as miR-200a, miR-21, miR-106b and miR-93, etc [29–31]. We therefore examined whether these miRNAs could be modulated by EYA2. The expression of these miRNAs was determined by quantitative PCR analysis. The results showed that EYA2 could up-regulate the levels of miR-93 and miR-106b in lung cancer cells (^*^p < 0.05, Figure 5A). Since the fold change affected by EYA2 was higher in miR-93 expression than in miR-106b expression, we selected miR-93 for subsequent studies. Next, we performed *in silico* analysis using the publicly available databases TargetScan. As shown in Figure 5B, the predicted binding sites for miR-93 in the 3′UTR of PTEN were conserved in different species. To determine whether PTEN is a direct target of miR-93, luciferase reporters containing either wild-type or mutant PTEN 3′UTRs were constructed. Luciferase reporter assay demonstrated that exogenous miR-93 repressed the luciferase activity controlled by wild-type PTEN 3′UTR, but not the luciferase activity controlled by mutant PTEN 3′UTR (^*^p < 0.05, Figure 5C). Moreover, Forced overexpression of miR-93 in A549 cells decreased PTEN expression at both the mRNA and protein levels (^*^p < 0.05, Figure 5D).

Furthermore, we tested whether EYA2 could repress PTEN expression by miR-93 upregulation. We suppressed endogenous miR-93 expression in A549 cells by transfection with anti-miR-93 oligonucleotides. The immunoblot assay showed that EYA2-mediated suppression of PTEN was attenuated by inhibition of endogenous miR-93 (Figure 5E). Consistently, the cell growth assay also showed that the cell growth advantage conferred by EYA2 was significantly compromised by inhibition of endogenous miR-93 (^*^p < 0.05, Figure 5F). In conclusion, EYA2 inhibited PTEN expression through modulation of miR-93.

### High expression of EYA2 predicts a poor prognosis in patients with lung cancer

**Figure 6:**
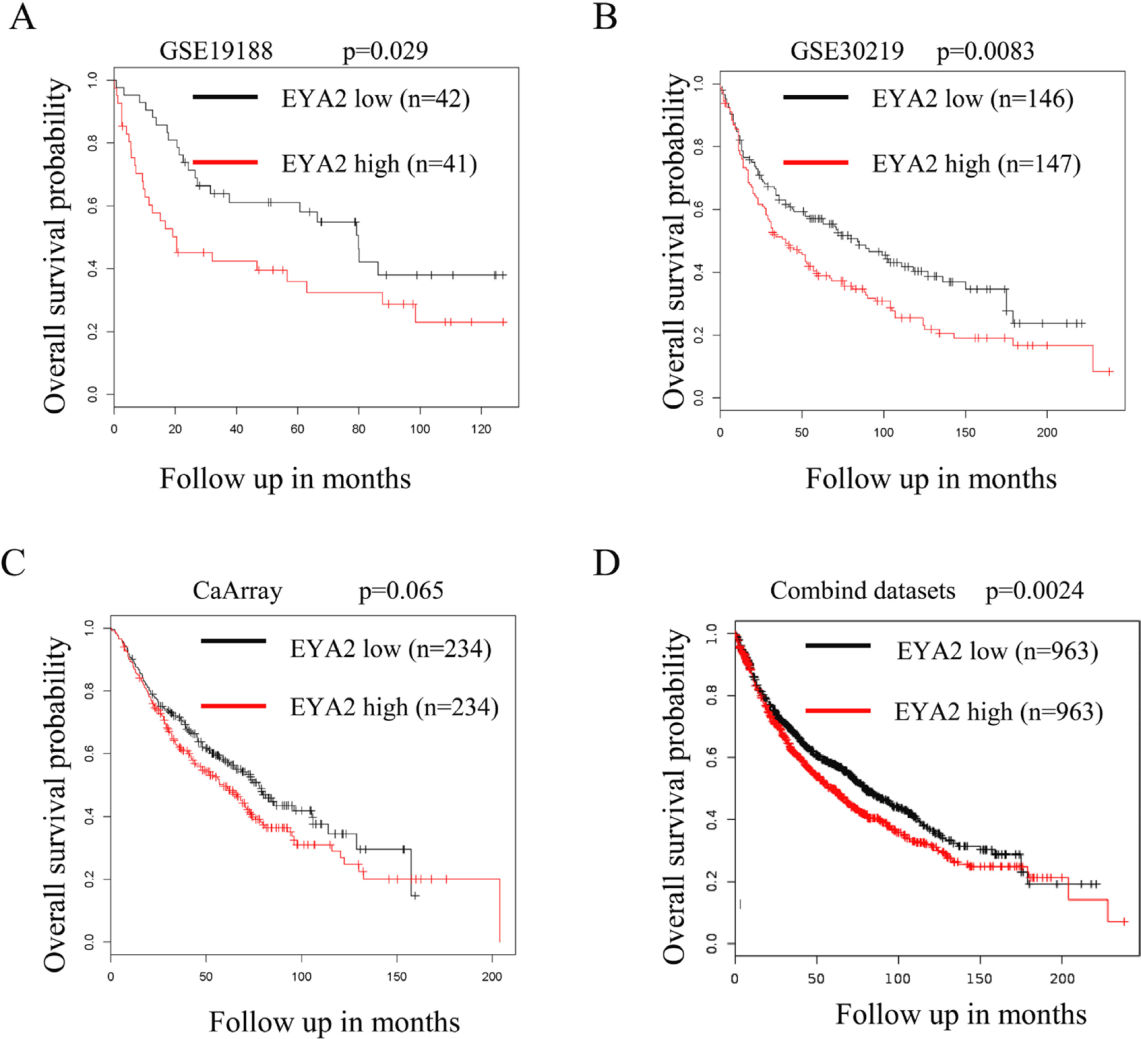
High expression of EYA2 predicts a poor prognosis in lung cancer The univariate analysis of survival analysis was performed using the Kaplan Meier plotter platform. Analysis of GSE19188 (**A**; *n* = 83, *p* = 0.029), GSE30219 (**B**; *n* = 293, *p* = 0.00839), CaArray lung cancer dataset (**C**; *n* = 468, *p* = 0.065) and the combinded lung cancer datasets (**D**; *n* = 1926, *p* = 0.0024) indicated that patients with high expression of EYA2 is associated with poor overall survival.

We analyzed the relationship between EYA2 expression and overall survival in patients with lung cancer. The univariate analysis of survival within the public available lung cancer datasets was performed using the Kaplan Meier plotter platform (http://kmplot.com) [32]. In the GSE19188 dataset, patients with lung cancer were divided into high (EYA2 high; *n =* 42) and low EYA2-expressing group (EYA2 low; *n =* 42). Kaplan Meier analysis revealed that lung cancer patients with high expression of EYA2 had a worse overall survival probability (Figure 6A; *p* = 0.029). Similarly, in the GSE30219 dataset, high level of EYA2 also conferred poor prognosis in lung cancer patients (Figure 6B; *p* = 0.0083). In the CaArray lung cancer dataset, there was trend toward inferior prognosis in patients with high expression of EYA2, even it did not reach a statistical significance (Figure 6C; *p* = 0.065). At last, we combined these public available lung cancer datasets and performed univariate analysis of survival using the Kaplan Meier plotter platform. Consistently, the results showed that patients with high expression of EYA2 had worse prognosis (Figure 6D; *p* = 0.0024). Collectively, these results suggest that high EYA2 expression was associated with poor overall survival in patients with lung cancer.

## DISCUSSION

The EYA family proteins are components of Retinal Determination Gene Network (RDGN) signaling, which is essential for the development of multiple organs in mammals by regulating cell proliferation, survival and differentiation [10, 13, 33, 34]. Aberrant expression of RDGN members such as DACH, EYA and SIX contributes to tumor initiation and progression [35]. The EYA family proteins have four members (EYA1-4), which can function as either highly conserved transcriptional coactivators or tyrosine phosphatases [35, 36]. Overexpression of EYAs has been found in various human cancers [35]. EYA1 is overexpressed in Wilms’ tumors [37], EYA2 is overexpressed in epithelial ovarian cancers and lung carcinoma [15, 18, 38], and EYA4 overexpression has been detected in MPNST (malignant peripheral nerve sheath tumor) [39]. What’s more, these EYA family proteins could facilitate tumor angiogenesis, cancer metastasis and were independent prognostic factors in several cancers [12, 18, 35, 40–44]. It has been reported that Eya2 is required to mediate the prometastatic functions of Six1 via the induction of TGF-β signaling, epithelial-mesenchymal transition, and cancer stem cell properties [15]; EYA and SIX1 are often co-overexpressed in tumors, and the SIX1-EYA2 interaction has been shown to be critical for metastasis in a breast cancer model [12, 15, 45]. Consistently with previous studies, this study showed that EYA2 was up-regulated in both small cell lung cancer and non-small cell lung cancer. In the subsequent experiment, our data demonstrated that EYA2 could promote lung cancer cell growth both *in vitro* an *in vivo*. Collectively, our findings suggest that EYA2 has an oncogenic role in the development of lung cancer.

The PI3K/AKT pathway is an intracellular signaling pathway important in regulating the cell growth, differentiation, survival and tumorigenesis [46–52]. PTEN acts as a critical tumor suppressor by inhibiting PI3K pathway [27]. The inactivation of PTEN by genetic (Loss of heterozygosity, mutations) or epigenetic regulation (promoter hypermethylation, miRNAs) has been found in various tumors [29, 53–55]. In the present study, we showed that EYA2 could suppress the PTEN expression via modulation of miR-93, which negatively regulated the expression of PTEN by binding to its 3′-UTR directly. Moreover, the cancer cell growth advantage conferred by EYA2 was significantly compromised via inhibition of endogenous miR-93. It is therefore safe to conclude that miR-93 is essential for EYA2 to repress the expression of PTEN in lung cancer cells.

Both *in vivo* and *in vitro* studies have indicated that EYAs could promote the proliferation and invasiveness of tumor cells [35]. On the contrary, removal of endogenous EYAs could prevent cancer metastasis [13, 15]. Here we found that EYA2 expression is significantly higher in tumor tissue compare to those in paired adjacent non-tumor tissues. Thus we speculate that inhibition of the EYAs activity could be a useful therapeutic strategy in lung cancer. Importantly, some compounds such as benzbromarone and benzarone have been identified as selective inhibitor series targeting the EYAs protein tyrosine phosphatase activity. It has been shown that these compounds have the ability to inhibit cell motility and tubulogenesis *in vitro*, and angiogenic sprouting *ex vivo*. As a result, the inhibition of EYA2 may represent a novel and promising treatment strategy for lung cancer.

In conclusion, overexpression of EYA2 leads to increased cell proliferation through suppression of PTEN in lung cancer, and EYA2 suppresses PTEN expression via upregulating miR-93. In addition, survival analysis indicates that lung cancer patients with high EYA2 expression have a worse overall survival. Our study not only yields a better understanding of the important role of EYA2 in lung cancer occurrence and progression, but also paves the way for the development of novel therapeutics target.

## MATERIALS AND METHODS

### Cell lines

A549, H1975 and H1650 cells (Cell bank of Chinese Academy of Sciences, Shanghai, China) were cultured in Dulbecco’s modified Eagle’s medium (Hyclone, Logan, UT, USA) supplemented with 10% fetal bovine serum (Hyclone, Logan, UT, USA), 0.1 mg/ml streptomycin, and 100 units/ml penicillin (Invitrogen, Carlsbad, CA, USA) in 5% CO_2_ atmosphere at 37°C.

### Tissue samples

Formalin-Fixed, Paraffin-Embedded (FFPE) lung cancer samples were collected at the First Affiliated Hospital of Zhengzhou University. The study was approved by the Research Ethics Committee of Zhengzhou University, and written informed consents were obtained from all patients who provided samples.

### Establishment of stable cell lines

Lentiviral production, titration, and infection were performed as previously described [56]. Briefly, lentiviral plasmids expressing EYA2 or vector control were co-transfected with pHelper plasmids in 293T cells. Lentiviral particles were harvested from the media after 48 hours of transfection, and purified with ultracentrifugation. Cells were then infected with lentiviruses encoding EYA2 or vector control. For knockdown of endogenous expression of EYA2, lentiviral constructs expressing EYA2 shRNAs were used. Cells were harvested at 72 hours after infection and the knockdown efficiency was evaluated by western blot analysis.

### RNA extraction and real-time PCR

Total RNA was isolated using the RNeasy mini kit (Qiagen, Germany). cDNA was prepared using the SuperScript^®^ III First-Strand Synthesis System (Invitrogen, Carlsbad, CA, USA). Quantitative PCR was performed using SYBR Green dye on an Applied Biosystems 7300 Real-time PCR system (Applied Biosystems, Foster City, CA, USA). The threshold cycle (C_T_) data was determinate using default threshold settings. The C_T_ is defined as the fractional cycle number at which the fluorescence passes the fixed threshold. The real-time PCR data was analysed by comparative C_T_ method [57].

### Western blot analysis

Western blot analysis was performed as previously described [58]. Briefly, cells were lysed in cold lysis buffer, proteins (20–30 μg) were resolved on SDS-PAGE, transferred onto PVDF membranes, and probed with antibodies for EYA2 (ab95875, Abcam), PTEN (ab31392, Abcam), and GAPDH (sc-32233, Santa Cruz Biotechnology) at 4°C overnight. Detection was performed with the SuperSignal West Femto Maximum Sensitivity Substrate Trial Kit (Pierce, Rockford, IL, USA). The band images were digitally captured and quantified with a FluorChem FC2 imaging system (Alpha Innotech, San Leandro, CA, USA). Protein levels were normalized to GAPDH and quantified with respect to vector control group.

### Immunohistochemistry

The FFPE sections were immunostained using the Dako EnVision™ Flex+ System (K8012; Dako, Glostrup, Denmark). Deparaffinization and epitope unmasking were carried out in a PT-Link using an EnVision™ Flex target retrieval solution (Dako, Carpinteria, CA, USA). The sections were treated with 0.3% hydrogen peroxide (H_2_O_2_) for 5 min to block endogenous peroxidase. Sections were incubated overnight at 4°C with the following antibodies: EYA2 (ab95875, Abcam), PTEN (ab31392, Abcam) and p-AKT (clone D9E, Cell Signaling Technology). The specimens were subsequently treated with EnVision™ Flex linker mouse or rabbit (15 min), EnVision™ Flex/HRP enzyme (30 min), and 3′3-diaminobenzidine tetrahydrochloride (10 min). The samples were counterstained with hematoxylin, dehydrated and mounted on a Richard-Allan Scientific Cyto seal XYL (Thermo Scientific, Waltham, MA, USA). The protein expression was scored semi-quantitatively based on the percentage of positive cells utilizing the following scale: +, <25%; ++, 25–49%; +++, 50–74%; and ++++, 75–100%.

### Cell proliferation and cell death assay

Cell proliferation was performed using Cell Counting Kit-8 (CCK-8, Dojindo, Tokyo, Japan) according to the manufacturer’s instructions. The absorbance value for each well was measured at 450 nm with a Multiskan FC microplate reader (Thermo scientific, Waltham, MA, USA). The fold change of cell numbers based on metabolic activity was calculated for each time-point [59]. Cell death was examined by Annexin V/PI fow cytometry assays as described previously [60]. All the experiments were performed in triplicate.

### Colony formation assay

Cells (2.0 × 10^3^) were seeded into 6-well plates in triplicate in 2 ml of complete growth medium. The medium was changed every three days. Two weeks later, cells were stained by 0.1% crystal violet (Sigma-Aldrich, St. Louis, MO, USA) in methanol for 10 min. Colonies (more than 50 μm diameter) were counted directly on the plate. All the experiments were performed in triplicate.

### PTEN 3′UTR reporter assays

Gene reporter assays were performed as previously reported [61]. Cells were co-transfected with synthetic miR-93, the wild-type or mutant 3′UTR of PTEN luciferase reporter vector pGL3-PTEN-3′UTR and pRL vector coding for the Renilla luciferase (Promega, Madison, WI, USA), and cells were then cultured for 48 to 72 hours. After that time, Cells were lysed with 500 μl 1×Passive Lysis Buffer (Promega) per well, incubated for 20 min at room temperature and cell debris removed by centrifugation. 50 μl supernatant was mixed with 50 μl of Dual-Glo reagent I (Promega) and firefiy luciferase luminescence measured in a GloMax^®^ 20/20 Luminometer (Promega, Madison, WI, USA). 50 μl Dual-Glo reagent II (Promega) was added and Renilla luciferase luminescence determined. Relative light units were calculated as Renilla/firefiy luminescence from triplicate wells and normalized to the negative control. All the experiments were performed in triplicate.

### Xenograft tumor assay

All procedures were conducted in accordance to Animal Care and Use Committee guidelines of Zhengzhou University. Mouse xenograft model were performed as previously reported [62]. Briefly, the BALB/c (6–8 weeks old) athymic nude mice were purchased from Vital River Laboratory Animal Technology (Charles River Laboratories, Beijing, China). The mice were randomly distributed into two groups and subcutaneously injected in the flank regions with 1.0 × 10^6^ /0.1 ml of PBS. Four weeks following implantation, mice were euthanized by asphyxiation in a CO_2_ chamber and tumors were excised and weighted.

### Microarray data analysis

EYA2 expression in lung cancer samples were analyzed using the Oncomine Cancer Microarray database (http://www.oncomine.org) as previously described [63]. Gene expression data were also obtained from NCBI Gene Expression Omnibus (GEO) database (Bhattacharjee’s dataset, GSE3398, GSE7670, GSE3268 and GSE19188). Expression data were log-transformed, median centered per array, and the standard deviation was normalized to one per array. The correlation analysis of EYA2 and PTEN were performed in GSE43580, GSE33532, ArrayExpress eumcu11 and TCGA dataset. The univariate analysis of survival analysis within the lung cancer data set of the GSE19188 (*n*  =  83), GSE30219 (*n =* 293) and CaArray (*n =* 468) was performed using the Kaplan Meier plotter platform (http://kmplot.com).

### Statistical analysis

Data were expressed as mean ± standard error of the mean (SEM). Between groups and among groups comparisons were conducted with Student *t*-test and ANOVA, respectively. Mann-Whitney *U*-test is used for nonparametric variables. EYA2 expression was analyzed by Fisher’s two-tailed exact test. Statistical analysis was performed using GraphPad Prism software version 4.0 (PRISM4) (GraphPad Software Inc, LaJolla, CA, USA) and the online statistics calculator VassarStats (www.vassarstats.net). *P*-values < 0.05 were considered statistically significant.

## SUPPLEMENTARY MATERIALS FIGURES AND TABLE



## References

[1] Hirsch FR, Suda K, Wiens J, Bunn PA (2016). New and emerging targeted treatments in advanced non-small-cell lung cancer. Lancet.

[2] Hiley CT, Le Quesne J, Santis G, Sharpe R, de Castro DG, Middleton G, Swanton C (2016). Challenges in molecular testing in non-small-cell lung cancer patients with advanced disease. Lancet.

[3] Hirsch FR, Scagliotti GV, Mulshine JL, Kwon R, Curran WJ, Wu YL, Paz-Ares L (2017). Lung cancer: current therapies and new targeted treatments. Lancet.

[4] Reck M, Heigener DF, Mok T, Soria JC, Rabe KF (2013). Management of non-small-cell lung cancer: recent developments. Lancet.

[5] Goldstraw P, Ball D, Jett JR, Le Chevalier T, Lim E, Nicholson AG, Shepherd FA (2011). Non-small-cell lung cancer. Lancet.

[6] van Meerbeeck JP, Fennell DA, De Ruysscher DK (2011). Small-cell lung cancer. Lancet.

[7] Li A, Wei ZJ, Ding H, Tang HS, Zhou HX, Yao X, Feng SQ (2017). Docetaxel versus docetaxel plus cisplatin for non-small-cell lung cancer: a meta-analysis of randomized clinical trials. Oncotarget.

[8] Qu J, Wang YN, Xu P, Xiang DX, Yang R, Wei W, Qu Q (2017). Clinical efficacy of icotinib in lung cancer patients with different EGFR mutation status: a meta-analysis. Oncotarget.

[9] Shtivelman E, Hensing T, Simon GR, Dennis PA, Otterson GA, Bueno R, Salgia R (2014). Molecular pathways and therapeutic targets in lung cancer. Oncotarget.

[10] Xu PX (2013). The EYA-SO/SIX complex in development and disease. Pediatr Nephrol.

[11] Rebay I (2015). Multiple Functions of the Eya Phosphotyrosine Phosphatase. Mol Cell Biol.

[12] Blevins MA, Towers CG, Patrick AN, Zhao R, Ford HL (2015). The SIX1-EYA transcriptional complex as a therapeutic target in cancer. Expert Opin Ther Targets.

[13] Tadjuidje E, Hegde RS (2013). The Eyes Absent proteins in development and disease. Cell Mol Life Sci.

[14] Zhang L, Yang N, Huang J, Buckanovich RJ, Liang S, Barchetti A, Vezzani C, O’Brien-Jenkins A, Wang J, Ward MR, Courreges MC, Fracchioli S, Medina A (2005). Transcriptional coactivator Drosophila eyes absent homologue 2 is up-regulated in epithelial ovarian cancer and promotes tumor growth. Cancer Res.

[15] Farabaugh SM, Micalizzi DS, Jedlicka P, Zhao R, Ford HL (2012). Eya2 is required to mediate the pro-metastatic functions of Six1 via the induction of TGF-beta signaling, epithelial-mesenchymal transition, and cancer stem cell properties. Oncogene.

[16] Ono R, Masuya M, Ishii S, Katayama N, Nosaka T (2017). Eya2, a Target Activated by Plzf, Is Critical for PLZF-RARA-Induced Leukemogenesis. Mol Cell Biol.

[17] Gao T, Zheng S, Li Q, Ran P, Sun L, Yuan Y, Xiao C (2015). Aberrant hypomethylation and overexpression of the eyes absent homologue 2 suppresses tumor cell growth of human lung adenocarcinoma cells. Oncol Rep.

[18] Liang Y, Xu X, Wang T, Li Y, You W, Fu J, Liu Y, Jin S, Ji Q, Zhao W, Song Q, Li L, Hong T (2017). The EGFR/miR-338-3p/EYA2 axis controls breast tumor growth and lung metastasis. Cell Death Dis.

[19] Yuan Y, Zheng S, Li Q, Xiang X, Gao T, Ran P, Sun L, Huang Q, Xie F, Du J, Xiao C (2016). Overexpression of miR-30a in lung adenocarcinoma A549 cell line inhibits migration and invasion via targeting EYA2. Acta Biochim Biophys Sin (Shanghai).

[20] Marsit CJ, Zheng S, Aldape K, Hinds PW, Nelson HH, Wiencke JK, Kelsey KT (2005). PTEN expression in non-small-cell lung cancer: evaluating its relation to tumor characteristics, allelic loss, and epigenetic alteration. Hum Pathol.

[21] Soria JC, Lee HY, Lee JI, Wang L, Issa JP, Kemp BL, Liu DD, Kurie JM, Mao L, Khuri FR (2002). Lack of PTEN expression in non-small cell lung cancer could be related to promoter methylation. Clin Cancer Res.

[22] Spoerke JM, O’Brien C, Huw L, Koeppen H, Fridlyand J, Brachmann RK, Haverty PM, Pandita A, Mohan S, Sampath D, Friedman LS, Ross L, Hampton GM (2012). Phosphoinositide 3-kinase (PI3K) pathway alterations are associated with histologic subtypes and are predictive of sensitivity to PI3K inhibitors in lung cancer preclinical models. Clin Cancer Res.

[23] Sos ML, Koker M, Weir BA, Heynck S, Rabinovsky R, Zander T, Seeger JM, Weiss J, Fischer F, Frommolt P, Michel K, Peifer M, Mermel C (2009). PTEN loss contributes to erlotinib resistance in EGFR-mutant lung cancer by activation of Akt and EGFR. Cancer Res.

[24] Yamamoto C, Basaki Y, Kawahara A, Nakashima K, Kage M, Izumi H, Kohno K, Uramoto H, Yasumoto K, Kuwano M, Ono M (2010). Loss of PTEN expression by blocking nuclear translocation of EGR1 in gefitinib-resistant lung cancer cells harboring epidermal growth factor receptor-activating mutations. Cancer Res.

[25] Endoh H, Yatabe Y, Kosaka T, Kuwano H, Mitsudomi T (2006). PTEN and PIK3CA expression is associated with prolonged survival after gefitinib treatment in EGFR-mutated lung cancer patients. J Thorac Oncol.

[26] Rhodes DR, Yu J, Shanker K, Deshpande N, Varambally R, Ghosh D, Barrette T, Pandey A, Chinnaiyan AM (2004). ONCOMINE: a cancer microarray database and integrated data-mining platform. Neoplasia.

[27] Perez-Ramirez C, Canadas-Garre M, Molina MA, Faus-Dader MJ, Calleja-Hernandez MA (2015). PTEN and PI3K/AKT in non-small-cell lung cancer. Pharmacogenomics.

[28] Ciuffreda L, Falcone I, Incani UC, Del Curatolo A, Conciatori F, Matteoni S, Vari S, Vaccaro V, Cognetti F, Milella M (2014). PTEN expression and function in adult cancer stem cells and prospects for therapeutic targeting. Adv Biol Regul.

[29] Li N, Miao Y, Shan Y, Liu B, Li Y, Zhao L, Jia L (2017). MiR-106b and miR-93 regulate cell progression by suppression of PTEN via PI3K/Akt pathway in breast cancer. Cell death & disease.

[30] Lasithiotaki I, Tsitoura E, Koutsopoulos A, Lagoudaki E, Koutoulaki C, Pitsidianakis G, Spandidos DA, Siafakas NM, Sourvinos G, Antoniou KM (2016). Aberrant expression of miR-21, miR-376c and miR-145 and their target host genes in Merkel cell polyomavirus-positive non-small cell lung cancer. Oncotarget.

[31] Cao L, Chen J, Ou B, Liu C, Zou Y, Chen Q (2017). GAS5 knockdown reduces the chemo-sensitivity of non-small cell lung cancer (NSCLC) cell to cisplatin (DDP) through regulating miR-21/PTEN axis. Biomed Pharmacother.

[32] Szasz AM, Lanczky A, Nagy A, Forster S, Hark K, Green JE, Boussioutas A, Busuttil R, Szabo A, Gyorffy B (2016). Cross-validation of survival associated biomarkers in gastric cancer using transcriptomic data of 1,065 patients. Oncotarget.

[33] Kumar JP (2009). The molecular circuitry governing retinal determination. Biochim Biophys Acta.

[34] Jemc J, Rebay I (2007). The eyes absent family of phosphotyrosine phosphatases: properties and roles in developmental regulation of transcription. Annu Rev Biochem.

[35] Liu Y, Han N, Zhou S, Zhou R, Yuan X, Xu H, Zhang C, Yin T, Wu K (2016). The DACH/EYA/SIX gene network and its role in tumor initiation and progression. Int J Cancer.

[36] Kong D, Liu Y, Liu Q, Han N, Zhang C, Pestell RG, Wu K, Wu G (2016). The retinal determination gene network: from developmental regulator to cancer therapeutic target. Oncotarget.

[37] Li CM, Guo M, Borczuk A, Powell CA, Wei M, Thaker HM, Friedman R, Klein U, Tycko B (2002). Gene expression in Wilms’ tumor mimics the earliest committed stage in the metanephric mesenchymal-epithelial transition. Am J Pathol.

[38] Huang YT, Heist RS, Chirieac LR, Lin X, Skaug V, Zienolddiny S, Haugen A, Wu MC, Wang Z, Su L, Asomaning K, Christiani DC (2009). Genome-wide analysis of survival in early-stage non-small-cell lung cancer. J Clin Oncol.

[39] Miller SJ, Lan ZD, Hardiman A, Wu J, Kordich JJ, Patmore DM, Hegde RS, Cripe TP, Cancelas JA, Collins MH, Ratner N (2010). Inhibition of Eyes Absent Homolog 4 expression induces malignant peripheral nerve sheath tumor necrosis. Oncogene.

[40] Emmett RA, Davidson KL, Gould NJ, Arasaradnam RP (2017). DNA methylation patterns in ulcerative colitis-associated cancer: a systematic review. Epigenomics.

[41] Lopez JI, Angulo JC, Martin A, Sanchez-Chapado M, Gonzalez-Corpas A, Colas B, Ropero S (2017). A DNA hypermethylation profile reveals new potential biomarkers for the evaluation of prognosis in urothelial bladder cancer. APMIS.

[42] Hao XY, Cai JP, Liu X, Chen W, Hou X, Chen D, Lai JM, Liang LJ, Yin XY (2016). EYA4 gene functions as a prognostic marker and inhibits the growth of intrahepatic cholangiocarcinoma. Chin J Cancer.

[43] Eisner A, Pazyra-Murphy MF, Durresi E, Zhou P, Zhao X, Chadwick EC, Xu PX, Hillman RT, Scott MP, Greenberg ME, Segal RA (2015). The Eya1 phosphatase promotes Shh signaling during hindbrain development and oncogenesis. Dev Cell.

[44] Krueger AB, Drasin DJ, Lea WA, Patrick AN, Patnaik S, Backos DS, Matheson CJ, Hu X, Barnaeva E, Holliday MJ, Blevins MA, Robin TP, Eisenmesser EZ (2014). Allosteric inhibitors of the Eya2 phosphatase are selective and inhibit Eya2-mediated cell migration. J Biol Chem.

[45] Fougerousse F, Durand M, Lopez S, Suel L, Demignon J, Thornton C, Ozaki H, Kawakami K, Barbet P, Beckmann JS, Maire P (2002). Six and Eya expression during human somitogenesis and MyoD gene family activation. J Muscle Res Cell Motil.

[46] Morgensztern D, McLeod HL (2005). PI3K/Akt/mTOR pathway as a target for cancer therapy. Anticancer Drugs.

[47] Fresno Vara JA, Casado E, de Castro J, Cejas P, Belda-Iniesta C, Gonzalez-Baron M (2004). PI3K/Akt signalling pathway and cancer. Cancer Treat Rev.

[48] Ballou LM, Lin HY, Fan G, Jiang YP, Lin RZ (2003). Activated G alpha q inhibits p110 alpha phosphatidylinositol 3-kinase and Akt. J Biol Chem.

[49] Chang F, Lee JT, Navolanic PM, Steelman LS, Shelton JG, Blalock WL, Franklin RA, McCubrey JA (2003). Involvement of PI3K/Akt pathway in cell cycle progression, apoptosis, and neoplastic transformation: a target for cancer chemotherapy. Leukemia.

[50] Massihnia D, Galvano A, Fanale D, Perez A, Castiglia M, Incorvaia L, Listi A, Rizzo S, Cicero G, Bazan V, Castorina S, Russo A (2016). Triple negative breast cancer: shedding light onto the role of pi3k/akt/mtor pathway. Oncotarget.

[51] Li X, Wu C, Chen N, Gu H, Yen A, Cao L, Wang E, Wang L (2016). PI3K/Akt/mTOR signaling pathway and targeted therapy for glioblastoma. Oncotarget.

[52] Markman B, Dienstmann R, Tabernero J (2010). Targeting the PI3K/Akt/mTOR pathway--beyond rapalogs. Oncotarget.

[53] Ronen S, Abbott DW, Kravtsov O, Abdelkader A, Xu Y, Banerjee A, Iczkowski KA (2017). PTEN loss and p27 loss differ among morphologic patterns of prostate cancer, including cribriform. Hum Pathol.

[54] Gu J, Ou W, Huang L, Wu J, Li S, Xu J, Feng J, Liu B, Zhou Y (2016). PTEN expression is associated with the outcome of lung cancer: evidence from a meta-analysis. Minerva Med.

[55] Cavazzoni A, La Monica S, Alfieri R, Ravelli A, Van Der Steen N, Sciarrillo R, Madeddu D, Lagrasta CAM, Quaini F, Bonelli M, Fumarola C, Cretella D, Digiacomo G (2017). Enhanced efficacy of AKT and FAK kinase combined inhibition in squamous cell lung carcinomas with stable reduction in PTEN. Oncotarget.

[56] Tian T, Li A, Lu H, Luo R, Zhang M, Li Z (2015). Six1 promotes glioblastoma cell proliferation and invasion by upregulation of connective tissue growth factor. Am J Cancer Res.

[57] Schmittgen TD, Livak KJ (2008). Analyzing real-time PCR data by the comparative C(T) method. Nat Protoc.

[58] Li Z, Lu L, Zhou Z, Xue W, Wang Y, Jin M, Qiu Y, Sun W, Fu X, Zhang X, Chang Y, Nan F, Yan J (2017). Recurrent mutations in epigenetic modifiers and the PI3K/AKT/mTOR pathway in subcutaneous panniculitis-like T-cell lymphoma. Br J Haematol.

[59] Quent VM, Loessner D, Friis T, Reichert JC, Hutmacher DW (2010). Discrepancies between metabolic activity and DNA content as tool to assess cell proliferation in cancer research. J Cell Mol Med.

[60] Tian T, Li A, Lu H, Luo R, Zhang M, Li Z (2015). TAZ promotes temozolomide resistance by upregulating MCL-1 in human glioma cells. Biochem Biophys Res Commun.

[61] Li Z, Tian T, Lv F, Chang Y, Wang X, Zhang L, Li X, Li L, Ma W, Wu J, Zhang M (2013). Six1 promotes proliferation of pancreatic cancer cells via upregulation of cyclin D1 expression. PloS one.

[62] Li Z, Tian T, Hu X, Zhang X, Li L, Nan F, Chang Y, Wang X, Sun Z, Lv F, Zhang M (2014). Targeting Six1 by lentivirus-mediated RNA interference inhibits colorectal cancer cell growth and invasion. Int J Clin Exp Pathol.

[63] Qiu X, Jiao J, Li Y, Tian T (2016). Overexpression of FZD7 promotes glioma cell proliferation by upregulating TAZ. Oncotarget.

