# Supplementary material for: EYA2 promotes lung cancer cell proliferation by downregulating the expression of PTEN
Source: Oncotarget. 2017 Dec 2;8(67):110837–48. doi: 10.18632/oncotarget.22860 (PMC5762288; doi:10.18632/oncotarget.22860)
Supplement: Supplementary file 1 [file oncotarget-08-110837-s001.pdf]

EYA2 promotes lung cancer cell proliferation by downregulating the expression of PTEN

SUPPLEMENTARY MATERIALS

Supplementary Table 1: The relationship between EYA2 expression and PTEN expression in lung cancer

		EYA4 expression		Coefficient of correlation (Phi)	*p* value
		+/++	+++/++++		
Total patients	PTEN expression				
	+/++	23	17	−0.28	0.017
	+++/++++	38	8		
NSCLC	PTEN expression				
	+/++	16	16	−0.32	0.012
	+++/++++	28	7		

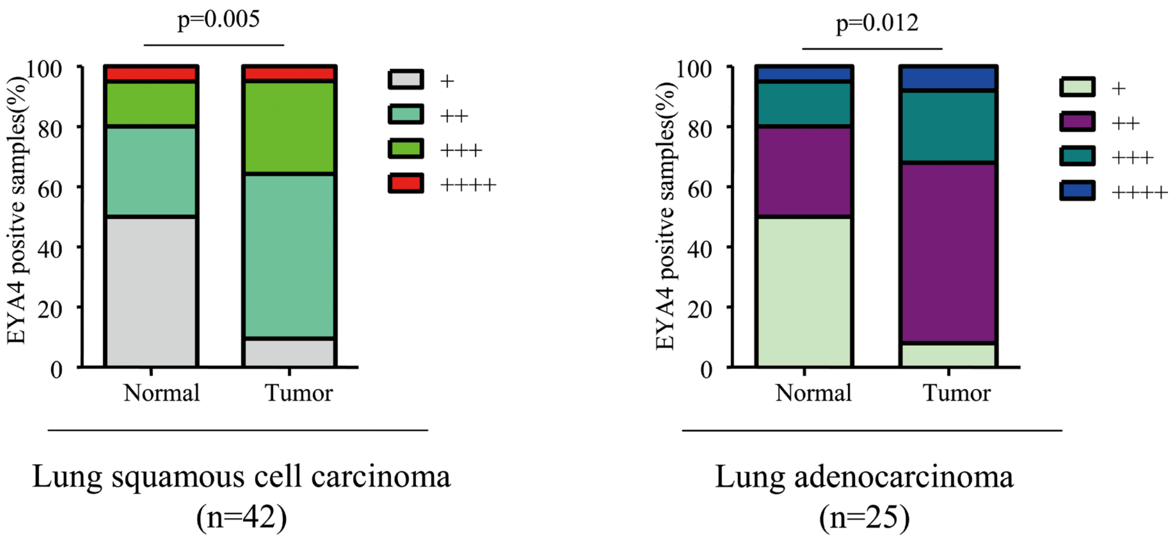

Supplementary Figure 1: EYA2 expression in lung cancer. EYA2 expression in lung squamous cell carcinoma (n = 42, p = 0.005) and lung adenocarcinoma (n = 25, p = 0.012) examined by immunohistochemisty. Expression levels of EYA2 were scored semi-quantitatively based on the percentage of positive cells according to the following scale: +, <25%; ++, 25–49%; +++, 50–74%; and ++++, 75–100%.

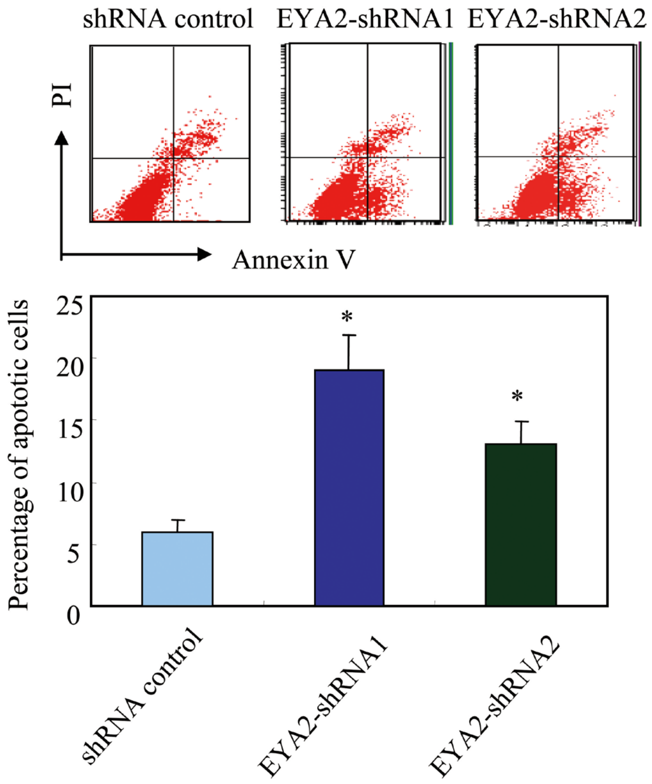

Supplementary Figure 2: Inhibition of EYA2 promoted the apoptosis of the A549 cells. Annexin V/PI staining assay of A549 cells stably transfected with EYA2 shRNAs (EYA2-shRNA1 or EYA2-shRNA2). All experiments were performed at least three times; bars, s.e.m.; $^{*}p < 0.05$.

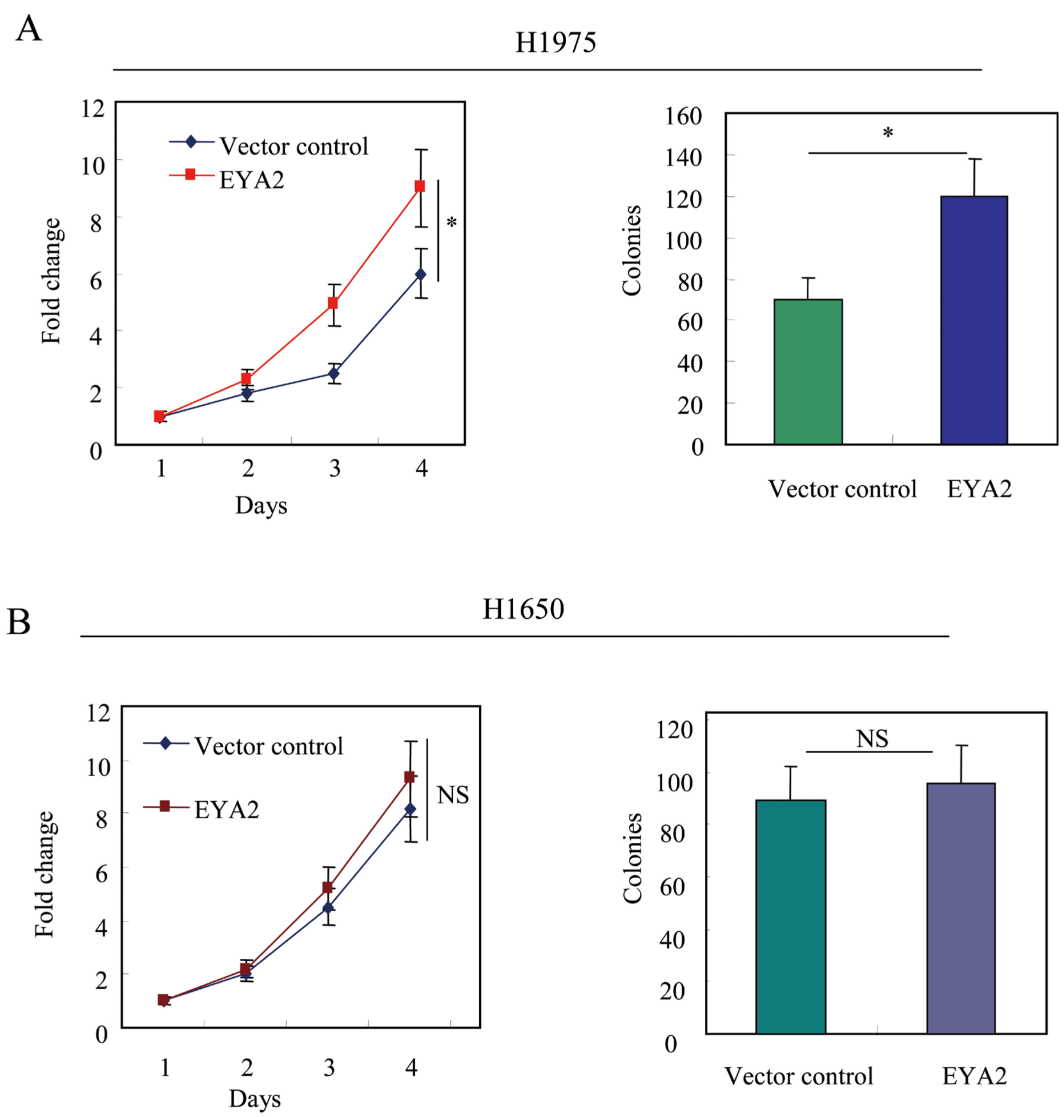

Supplementary Figure 3: Overexpression of EYA2 promoted cell proliferation. (**A**, **B**) cell growth assay (A) and colony formation assay (B) of H1975 cells with overexpression of EYA2. (**C**, **D**) cell growth assay (C) and colony formation assay (D) in a PTEN null cell line H1650 after overexpression of EYA2. All experiments were performed at least three times; bars, s.e.m.; $^{*}p < 0.05$; NS, not significant.
